# Optimization of Phenolic Compounds Extraction from Aerial Parts of *Fabiana punensis* S. C. Arroyo by Ultrasound- and Microwave-Assisted Extraction

**DOI:** 10.3390/molecules29153578

**Published:** 2024-07-29

**Authors:** Daniela Alejandra González, José Martínez Chamás, María Eugenia Orqueda, Mariana Leal, Agostina Conta, María Inés Mercado, María Inés Isla, Iris Catiana Zampini

**Affiliations:** 1 Instituto de Bioprospección y Fisiología Vegetal (INBIOFIV-CONICET-UNT), San Martín 1545, San Miguel de Tucumán 4000, Tucumán, Argentina; danigonz37@gmail.com (D.A.G.); eorqueda@yahoo.com.ar (M.E.O.); maariileal@hotmail.com (M.L.); agosconta20@gmail.com (A.C.); misla@csnat.unt.edu.ar (M.I.I.); 2 Facultad de Ciencias Naturales e IML, Universidad Nacional de Tucumán, Miguel Lillo 205, San Miguel de Tucumán 4000, Tucumán, Argentina; 3 Instituto de Morfología Vegetal, Área Botánica, Fundación Miguel Lillo, Miguel Lillo 251, San Miguel de Tucumán 4000, Tucumán, Argentina; mimercado@lillo.org.ar

**Keywords:** Solanaceae, *Fabiana punensis*, medicinal plants, optimization, RSM, anti-inflammatory, antioxidant, antimutagenic

## Abstract

*Fabiana punensis* S. C. Arroyo is a subshrub or shrub that is indigenous to the arid and semiarid region of northern Argentina and is known to possess several medicinal properties. The objective of this study was to optimize the extraction conditions so as to maximize the yield of bioactive total phenolic compound (TPC) and flavonoids (F) of *F. punensis’* aerial parts by using non-conventional extraction methods, namely ultrasound-assisted extraction, UAE, and microwave-assisted extraction, MAE, and to compare the biological activities and toxicity of optimized extracts vs. conventional extracts, i.e., those gained by maceration. Response Surface Methodology (RSM) was used to apply factorial designs to optimize the parameters of extraction: solid-to-liquid ratio, extraction time, ultrasound amplitude, and microwave power. The experimental values for TPC and F and antioxidant activity under the optimal extraction conditions were not significantly different from the predicted values, demonstrating the accuracy of the mathematical models. Similar HPLC-DAD patterns were found between conventional and UAE- and MAE-optimized extracts. The main constituents of the extracts correspond to phenolic compounds (flavonoids and phenolic acids) and apigenin was identified. All extracts showed high scavenger capacity on ABTS^•+^, O_2_^•−^ and H_2_O_2_, enabling the inhibition of the pro-inflammatory enzymes xanthine oxidase (XO) and lipoxygenase (LOX). They also showed an antimutagenic effect in *Salmonella* Typhimurium assay and cytotoxic/anti-proliferative activity on human melanoma cells (SKMEL-28). Toxicological evaluation indicates its safety. The results of this work are important in the development of efficient and sustainable methods for obtaining bioactive compounds from *F. punensis* for the prevention of chronic degenerative diseases associated with oxidative stress, inflammation, and DNA damage.

## 1. Introduction

*Fabiana punensis* S. C. Arroyo (Solanaceae) is a subshrub or shrub and grows primarily in the subalpine or subarctic biome [1]. In northern Argentina it is found in arid and semiarid regions and is known to possess several medicinal properties [2]. The biological activities of *F. punensis* are attributed to its composition of phenolic compounds [2]. *F. punensis* and other species belonging to the Solanaceae family have also been employed as medicine, building material, forage, fuel and elements in spiritual activities [2]. The resinous exudates from *Fabiana* leaves and branches are used in traditional medicine to immobilize fractured extremities, while their infusion and decoction are used as antiseptic and anti-inflammatory agents [2]. To our knowledge, there are few reports that validate their popular use [2,3].

Due to the antioxidant, anticancer, antibacterial, and anti-inflammatory activities exerted by phenolic compounds present in plants, they have gained attention as potential candidates for pharmacological use. Hence, the extraction process plays a pivotal role in the recovery of these bioactive compounds. However, optimization is necessary to improve the extraction yield, considering both the chemical composition and biological activities [4]. Enhancing the extraction yields of natural bioactive compounds, like phenolics, might modify their functional properties [5]; therefore, understanding how solvents, temperature, and extraction time have a direct impact on the release of phenolic compounds and their bioactivity is essential [6]. Several phenolic compound extraction methods have been reported, assessed, and classified as conventional and non-conventional. The standard conventional procedures for phenolic extraction include stirring, maceration, infusion, cold pressing, and hydro-distillation, among others. Advanced extraction methods such as microwave-assisted extraction (MAE), ultrasound-assisted extraction (UAE), high pressure-assisted extraction (HPAE), high voltage electric discharge-assisted extraction (HVED), pulsed electric field-assisted extraction (PEF), and supercritical fluid extraction (SFE) were developed [7,8]. These methods save energy and chemicals and may be applied on a large scale [8]. Among them, UAE is an environmentally friendly and economically viable alternative to conventional techniques for food and natural products; this procedure relies on the cavitation effect, results in both physical and mechanical damage to the plant matrix, and improves extraction recovery. In addition, this extraction method consumes less solvent and energy, and has shorter extraction time and higher product recovery rates compared to conventional methods [9]. On the other hand, MAE involves heating water molecules within the plant matrix, causing the cell walls to expand and heat up, breaking down, and releasing internal material and components from the cells [10]. However, unconventional methods should be optimized for the target compounds by using advanced statistical tools [11]. Response surface methodology (RSM) is a statistical procedure that simultaneously solves or determines the response to a multivariate problem by using quantitative data from an appropriate experimental design [12]. In contrast to the traditional empirical approach, RSM can provide a mathematical model, adjust for any interactions between test variables, and reduce the number of trials [13].

The aim of the present study was to optimize the extraction conditions to maximize the yield of bioactive total phenolic compound (TPC) and flavonoids (F) of *F. punensis*’ aerial parts by using non-conventional extraction methods, namely ultrasound-assisted extraction, UAE, and microwave-assisted extraction, MAE, as well as to compare the biological activities and toxicity of optimized extracts vs. conventional extracts, i.e., those gained by maceration.

## 2. Results

### 2.1. Non-Conventional Extraction Optimizations

#### 2.1.1. Ultrasound Assisted Extraction (UAE)

The efficiency of ultrasound-assisted extraction from aerial parts of *F. punensis* under the influence of three variables, namely, ultrasound amplitude, time and solid-to-liquid ratio, on three levels, that is, 25, 50 and 100% amplitude; 10, 20 and 30 min and 0.25, 0.50 and 1 g of plant material per 20 mL of solvent, was studied. According to the design for optimization, 32 experiments were performed. Phenolic compounds and flavonoid contents of each obtained extract and their ABTS^•+^ scavenging activity were determined; Supplementary Table S1 shows the results. The data were analyzed by multiple regression, and the coefficients of the third polynomial order model were calculated (Supplementary Table S2). Significant coefficients were used to develop the prediction models for the TPC and F response values. However, the ABTS radical cation (ABTS^•+^) scavenging values could not be modeled as they did not fit the data. The predicted statistical models fitted F-values of 109.79 and 32.31 for TPC and F, respectively, denoting the significance of the models. The R^2^ values (0.98 and 0.94) suggested the accurate choice of the statistical models for both variables.

The reduced cubic models obtained by Central Composite Design (CCD) for the optimization of TPC (1) and F (2) extraction by UAE are shown below, with X_1_ (amplitude), X_2_ (time) and X_3_ (solid/liquid ratio):

Y_TFC_ = 661.05 − 6.64 X_1_ − 3.86 X_2_ + 234.55 X_3_ − 11.67 X_1_X_2_ − 16.70 X_1_X_3_ + 129.71 X_2_X_3_ − 15.85 X_2_^1^ + 53.91 X_3_^2^ + 55.75 X_1_X_2_X_3_ + 132.41X_1_^2^ + 104.14 X_1_^2^X_3_ (1)

Y_F_ = 108.03 − 11.78 X_1_ − 5.35 X_2_ + 8.60 X_3_ − 3.57 X_1_X_2_ − 5.25 X_1_X_3_ + 9.79 X_2_X_3_ − 7.03 X_1_^2^ − 11.01 X_3_^2^ + 11.30 X_1_^2^X_2_ + 37.07 X_1_^2^X_3_ (2)

The terms X_3_ (solid/liquid ratio), X_3_^2^ (solid/liquid ratio^2^), X_2_X_3_ (time × solid/liquid ratio) and X_123_ (amplitude × time × solid/liquid ratio) (*p* ≤ 0.05) showed significant effects on the two modeled variables, demonstrating the importance of the factor solid/liquid ratio for this extraction process. Likewise, amplitude per se affected negatively the extraction yield of both TPC and F, with X_1_ and X_1_^2^ factors with values of *p* ≤ 0.05 (Supplementary Table S2).

##### Analysis of Response Surface Plots for UAE

**Figure 1:**
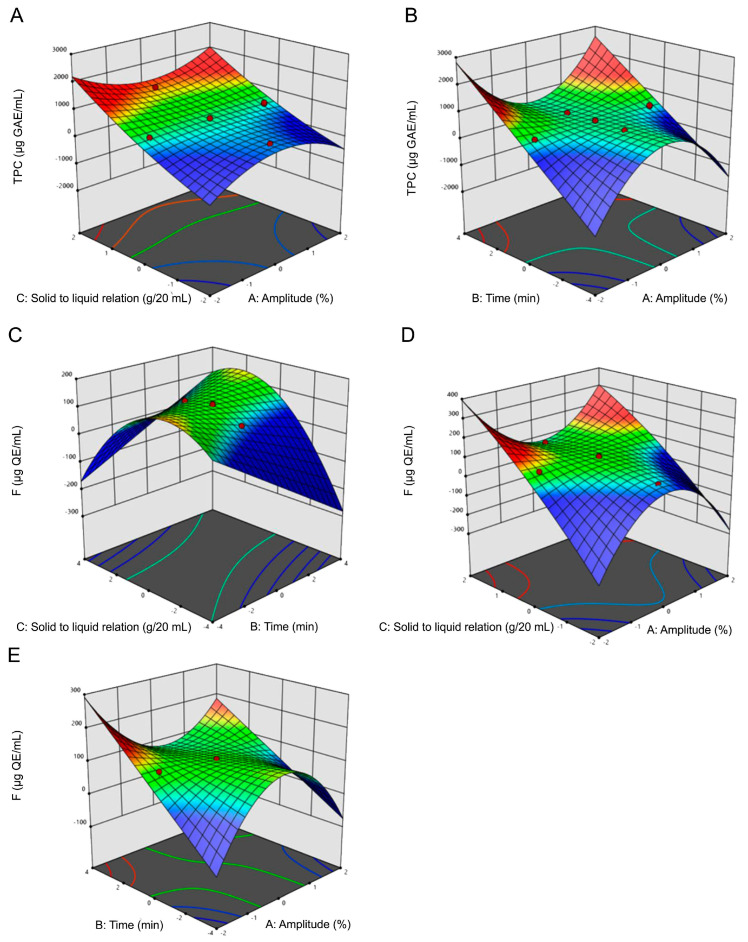
Response surface plots of ultrasound-assisted extraction (UAE) of total phenolic compounds (TPC) and flavonoids (F). Influence on TPC extraction of (**A**) solid/liquid ratio vs. UAE amplitude; (**B**) UAE time vs. amplitude. Influence on F content of (**C**) solid/liquid ratio vs. UAE time; (**D**) Solid/liquid ratio vs. UAE amplitude; and (**E**) Time vs. UAE amplitude. Blue and red areas represented low and high yields of TPC and F concentration, respectively.

The synergetic effect of the selected three factors on TPC and F yield was shown in Figure 1. One factor was fixed at 0 level, and the impact of the other two factors on the response value was analyzed. Figure 1A–B showed the effect of factor interactions on TPC content, and Figure 1C–E exhibited the factor effects over F content. In these figures, blue and red areas represented low and high yields of TPC and F concentration, respectively. TPC and F extraction yield increased with the increase of the ultrasonic time. The maximum TPC content was detected at level 1 (100%) of the amplitude factor, whereas for F it was observed at level −1 (25%) amplitude.

##### Optimal Extraction Conditions for UAE

Optimal extraction conditions to maximize the yield of TPC and F by UAE were 25% ultrasound amplitude, with time at 30 min and a solid/liquid ratio of 1:20 (Table 1). The experimental values of TPC and F yield obtained at optimum conditions showed good agreement with the values theoretically predicted by the model, with a prediction error ranging from 5 to 7%. Up to date, no studies have optimized extractions using non-conventional methods for the *Fabiana* species.

#### 2.1.2. Microwave Assisted Extraction (MAE)

The efficiency of microwave-assisted extraction from aerial parts of *F. punensis* under the influence of three variables, i.e., power, time and solid-to-liquid ratio, on three levels, i.e., power of 100, 300 and 500 W; 10, 20 and 30 min; and 0.25, 0.50 and 1 g of plant material per 20 mL of solvent, were studied. According to the design for optimization, 32 experiments were performed (Supplementary Table S3). The data on yield for TPC and F and ABTS^•+^ scavenging percentages were evaluated by multiple regression analyses. The regression coefficients of the third-order polynomial models were calculated, allowing the modeling of the response variables, and were used to develop the models for predicting the response values (Supplementary Table S4). The regression result equations obtained by CCD for the optimization of the extraction of TPC (3), F (4) and antioxidant activity (5) by MAE are shown below, with X_1_ (power), X_2_ (time) and X_3_ (solid/liquid ratio):

Y_TPC_ = 366.55 + 11.61 X_1_ + 0.1309 X_2_ + 102.88 X_3_ + 14.99 X_1_X_2_ + 3.81 X_1_X_3_ + 1.49 X_1_^2^X_3_ (3)

Y_F_ = 79.79 + 0.1028 X_1_ + 34.49 X_3_ − 2.21X_1_X_3_ + 1.88 X_1_X_2_ + 4.22 X_3_^2^ + 11.62 X_1_^2^X_3_ (4)

Y_ABTS_ = 27.04 + 0.29 X_1_ + 1.67 X_2_ + 3.8 X_3_ + 2.01 X_1_X_2_ − 0.194 X_1_X_3_ + 1.98 X_2_X_3_ − 0.20 X_1_^2^ + 13.46 X_1_^2^X_3_ (5)

These models were adjusted for the three modeled variables (TPC, F and ABTS^•+^ scavenging) with R^2^ 0.9609, 0.9610 and 0.9306, respectively.

##### Analysis of Response Surface Plots for MAE

**Figure 2:**
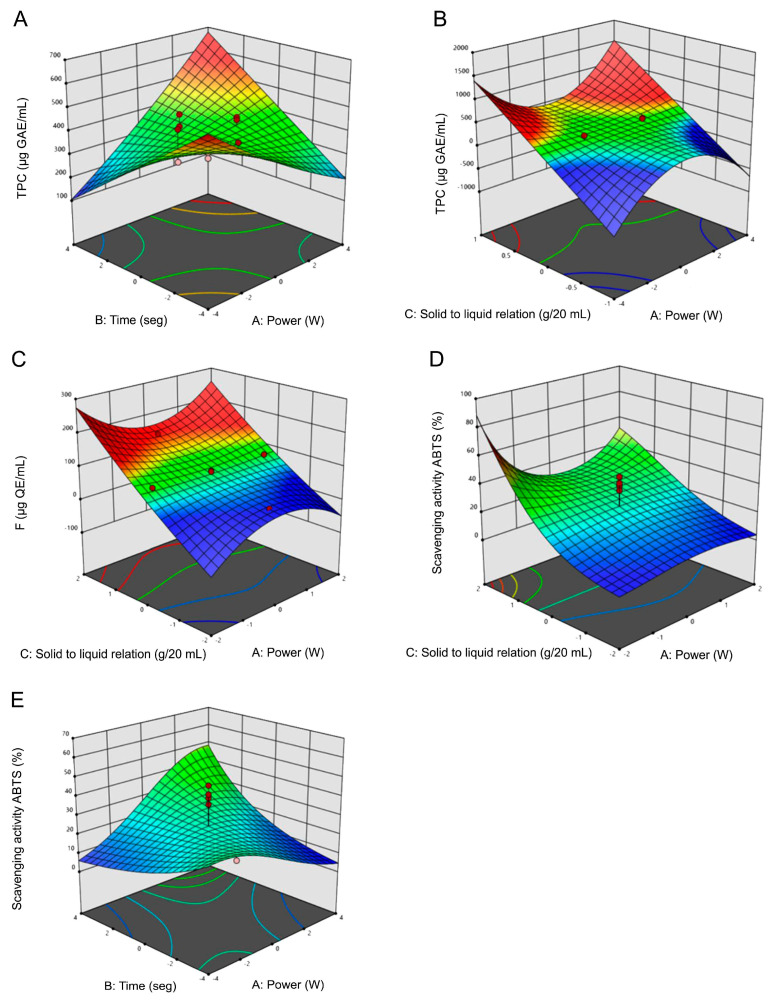
Response surface plots of microwave assisted extraction (MAE) of total phenolic compounds (TPC), flavonoids (F) and scavenging of ABTS^•+^ percentages. Influence on TPC extraction of (**A**) time vs. MAE Power, (**B**) solid/liquid ratio vs. MAE Power. Influence on F of (**C**) solid/liquid ratio vs. MAE Power. Influence on scavenging activity of ABTS^•+^, (**D**) solid/liquid ratio vs. MAE Power, (**E**) time vs. MAE Power. Blue and red areas represented low and high yields of TPC and F concentration or scavenging activity of ABTS^•+^, respectively.

RSM graphs (Figure 2) reveal a direct relationship between the TPC yield and the levels of time and solid/liquid ratio. Similarly, an increase in power also corresponds to higher extraction yields. However, it is noteworthy that an increase in power enhances yields only when accompanied by a simultaneous increase in the solid/liquid ratio. Significant increases in the concentration of F compounds were noted with escalating levels of the solid/liquid ratio. Through the model’s predictive terms, a positive linear relationship was shown between F concentration and the factors power and solid/liquid ratio. However, the interaction between both factors (power × solid/liquid ratio) exhibited a negative correlation with F concentration, as the model coefficients indicated. In the analysis of ABTS^•+^ scavenging, notable increments in scavenging percentages corresponded to increasing levels in the solid/liquid ratio factor and extraction times, as depicted in Figure 2D. Additionally, when the solid/liquid ratio factor remained at its mean level, the response variable exhibited no fluctuations despite variations in the power and time factors. The power factor exhibited a moderate impact on the extraction of antioxidant compounds, along with the influential interactions of power and time (X_1_X_2_) as well as time and solid/liquid ratio (X_2_X_3_). However, the interaction of power and solid/liquid ratio (X_1_X_3_) negatively affected extraction yield, requiring more plant material as power increased to augment extraction yields. Solid/liquid ratio had a significant influence on the three variables, including the quadratic interaction of power and the solid/liquid ratio (X_12_X_3_).

The maximum TPC content was detected at level 1 (700 W) of the power factor at the maximum extraction time (90 s) and at 1:20 (g/mL) solid/liquid ratio. The same applies to scavenging ABTS^•+^ percentage. However, the highest F yield was reached at level 0 of the factors power and time (500 W and 60 s, respectively). In addition to the influence of the factor solid/liquid ratio, the interactions of power^2^ × solid/liquid ratio factors, also had a significant influence, which may be related to a higher speed of the diffusion process due to the increase in temperature.

##### Optimal Extraction Conditions for MAE

Optimal extraction conditions to maximize the yields of TPC and F and improve the values of the ABTS^•+^ scavenging percentage of F. *punensis* by MAE were 700 W power for 90 s and a solid/liquid ratio of 1:20 (Table 1). Experimentally obtained values for the TPC and F content and scavenging ABTS^•+^ percentages of the optimized extracts showed as being aligned with the theoretically predicted values, with a prediction error of 2–7%, demonstrating the accuracy of the models in fitting the variable responses under optimal MAE conditions.

### 2.2. Comparisons of the Main Characteristics of Conventional Maceration vs. the Optimized Extracts Obtained by Non-Conventional Methods (UAE and MAE)

In order to compare the extraction efficiency of the methods, the values from each response variable were compared at the optimum conditions and at the 3 levels of each factor, keeping the factors time and solid/liquid ratio constant at their maximum level. TPC and F content when using either UAE or conventional maceration was not significantly different; however, the optimized extract obtained by MAE showed a significantly lower yield of TPC and F (Table 2). Although the extraction performed with UAE showed a concentration of TPC and F similar to conventional maceration, it was achieved in a period 300 times shorter. Concurrently, the concentration of soluble principles (DW) increased significantly. The percentage of scavenging activity of ABTS^•+^ was significantly higher in the optimized extract attained by UAE. However, despite extracting lower TPC content, the one attained by MAE presented similar antioxidant activity to conventional maceration. This similarity was also observed in the content of soluble principles obtained in the traditional maceration and the extraction obtained by MAE. It is important to highlight that although the MAE-optimized extract yielded significantly lower amounts of TPC and F, the time taken to do the extraction was up to 6000 times shorter compared to the traditional maceration. Furthermore, MAE extracts showed enrichment in F content (Table 2).

### 2.3. Scanning Electron Microscopy of Plant Material Subjected to Different Extraction Methods

**Figure 3:**
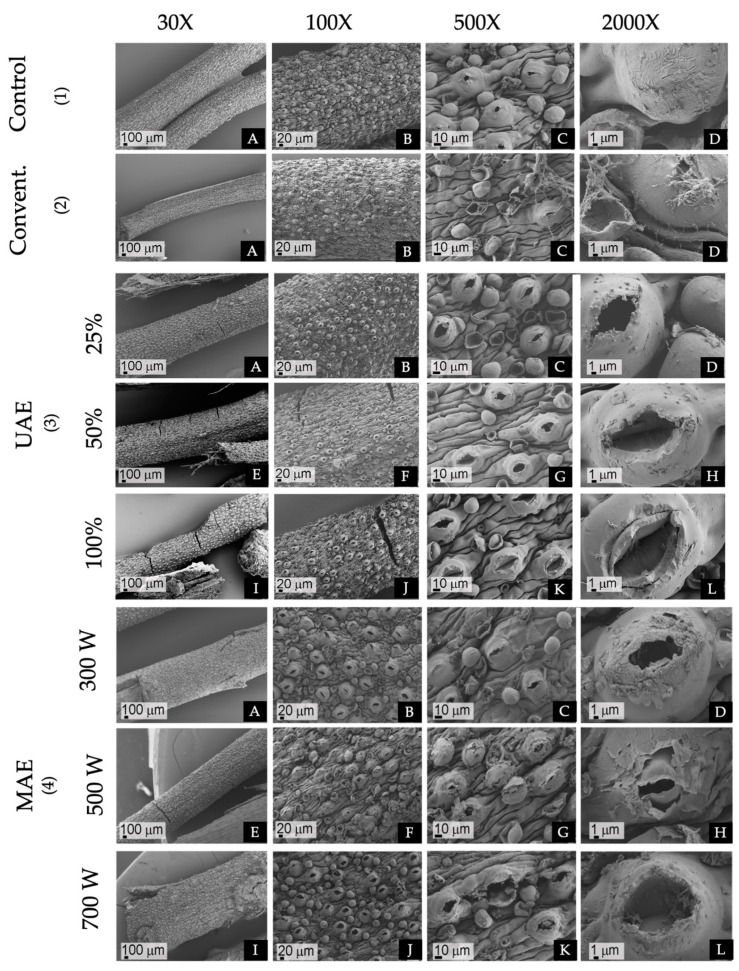
SEM of the material of *F. punensis*. (1)(**A**–**D**) Rehydrated in physiological solution, (2)(**A**–**D**) Exposed to conventional maceration (Convent), (3)(**A**–**D**) Exposed to UAE (ultrasound-assisted extraction) 25% of amplitude, (**E**–**H**) 50% of amplitude and (**I**–**L**) 100% of amplitude, (4)(**A**–**D**) exposed to MAE (microwave assisted extraction) at 300 W of power, (**E**–**H**) 500W of power and (**I**–**L**) 700 W of power. Capture taken at 30×, 100×, 500× and 2000×.

Dried plant material of *F. punensis* rehydrated in physiological solution (Figure 3(1A–D)) was used for comparison with the plant material exposed to the different extraction methods. The traditional maceration did not result in significant structural alterations. Tissue integrity was kept within the plant matrix while the surface exhibited a rough texture characterized by thin filaments on the cuticles, likely originating from the internal tissues via slightly opened ostioles or from collapsed glandular trichomes (Figure 3 (2A–D)). In contrast, exposure to increasing levels of amplitude of UAE induced notable structural changes (Figure 3(3A–L)). These alterations included cracking, wrinkling, and fragmentation of cell walls, with stomata hypertrophy and ostiole dilation, particularly evident at the highest amplitudes (Figure 3(3I–L)). Although cuticle integrity was kept, increased amplitude and cavitation led to micropore formation, probably due to trichome collapse and detachment (Figure 3(3L)). Similarly, escalating levels of MAE power resulted in progressive cracking and rupture of the plant material (Figure 3(4A,E,I)). The SEM analysis revealed widened stomatal ostioles, with pronounced glandular trichome collapse and cuticle detachment at the highest energy intensities (Figure 3(4C,D,G,H,K)).

### 2.4. HPLC-DAD of Conventional and UAE- and MAE-Optimized Extracts from F. punensis

**Figure 4:**
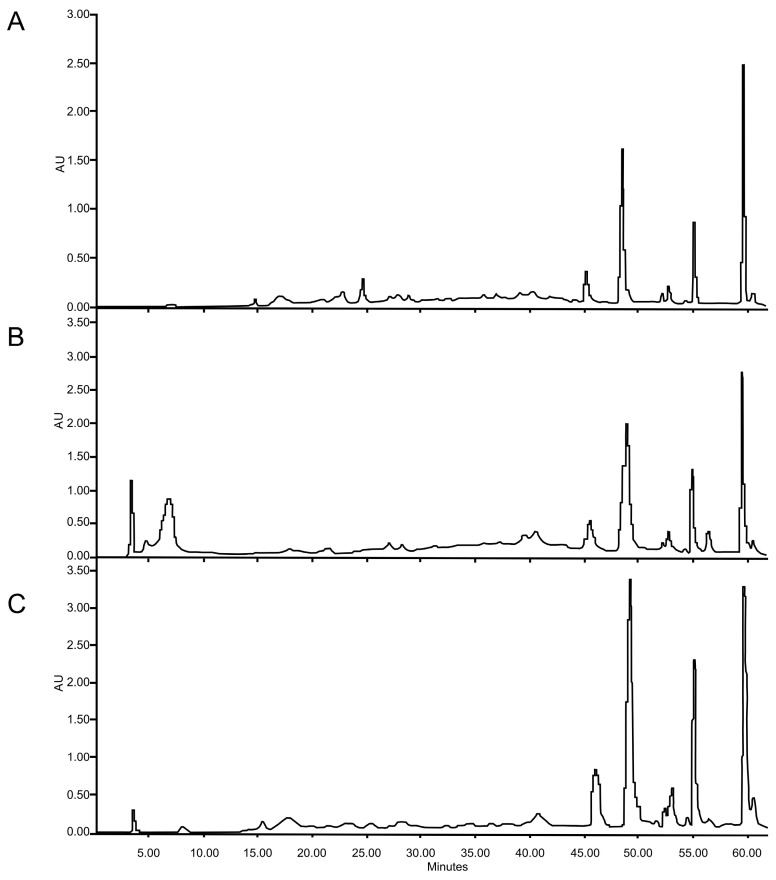
HPLC-DAD profiles of the extracted of *F. punensis* obtained by (**A**) conventional maceration; (**B**) ultrasound-assisted extraction (UAE) and (**C**) microwave-assisted extraction (MAE). The fingerprints were registered at 330 nm.

Similar HPLC profiles were found in conventional and UAE- and MAE-optimized extracts (Figure 4) showing four major peaks at retention times (R_t_) of 46, 49, 55, and 60 min. The peak at 46 min was identified as apigenin according to UV spectra [14] and co-chromatography with a standard. The other compounds have not been identified, but they are phenolic compounds, and the peaks with R_t_ of 49 and 55 min exhibit UV spectra corresponding to flavonoid traits [14], while the peak with 60 min retention time presents UV band characteristics of phenolic acid. Regarding the intensity of the peaks, conventional maceration, as well as the extract obtained with ultrasound, presents similar intensity, the peak with R_t_ 60 min being the predominant one. In contrast, the extracts obtained by MAE, have higher peak intensities. Additionally, the relationship between the peaks in the MAE extract is different; in this case, the peak at R_t_ 49 min increases and its intensity is higher than the peak at 60 min, whereas the peak at Rt 55 also showed an increase, which shows that this extract would be enriched with flavonoids.

### 2.5. In Vitro Biological Activities

#### 2.5.1. Anti-inflammatory Activity of *F. punensis* Extracts. Lipooxygenase (LOX) and Xanthine Oxidase (XO) Inhibition

**Figure 5:**
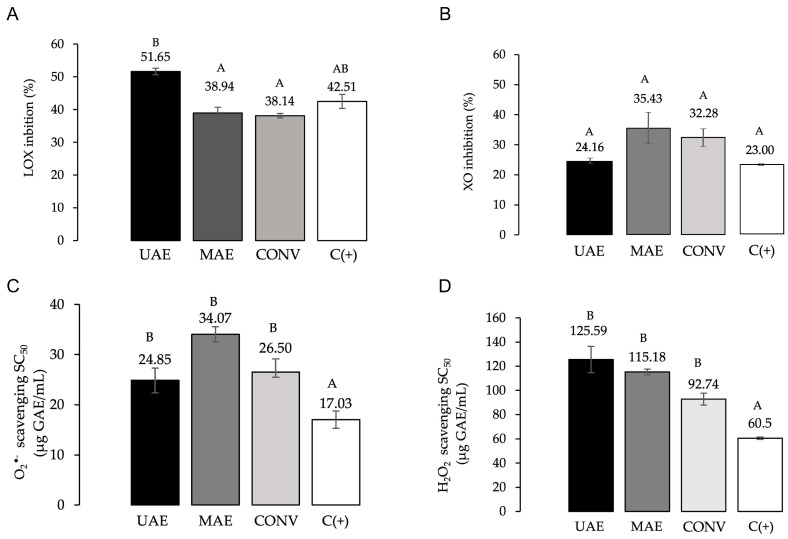
Anti-inflammatory and antioxidant properties of the extracts from *F. punensis*: conventional maceration (CONV), ultrasound-assisted extraction (UAE) and microwave-assisted extraction (MAE). (**A**) LOX activity. (**B**) XO activity. (**C**) Scavenging activity of O_2_^•−^ and (**D**) Scavenging activity of H_2_O_2_. GAE: gallic acid equivalents. Naproxen (25 µg/mL) and allopurinol (1 µg/mL) were used as reference inhibitors of LOX and XO enzymes, respectively (C+). Quercetin was included as antioxidant positive control. Values were expressed as the mean of three determinations ± standard deviation. Different letters above the bars represent statistically significant differences at *p* ≤ 0.05, according to Tukey’s test.

Extracts of *F. punensis* obtained by conventional and non-conventional methods inhibited the LOX enzyme activity (Figure 5A), with inhibition percentages of 38.14 to 51.65%. All extracts were evaluated in equal concentrations (12.5 µg GAE/mL); the extract obtained by UAE showed the highest activity, actually similar to naproxen, a commercial anti-inflammatory compound.

In addition, all the extracts of *F. punensis* at concentration 25 µg GAE/mL inhibited the activity of XO between 24.16 and 35.43%, without significant differences between the samples (Figure 5B). The activity was similar to that exerted by 1 µg/mL of the positive control, allopurinol, a synthetic inhibitor of XO, and although the positive control was active at a low concentration, it should be taken into account that it is a pure compound.

#### 2.5.2. Antioxidant Properties of *F. punensis* Extracts. Superoxide Anion Radical (O_2_^•−^) Scavenging and Hydrogen Peroxide (H_2_O_2_) Scavenging

The three *F. punensis* extracts evaluated scavenged the O_2_^•−^, with SC_50_ values ranging 92.74–125.59 µg GAE/mL (Figure 5C).

In terms of H_2_O_2_ scavenging activity, SC_50_ values were between 24.85 and 34.07 µg GAE/mL (Figure 5D). No significant differences were found between the extracts obtained by both conventional and non-conventional methods. The SC_50_ values obtained for the compound used as reference, quercetin, were significantly lower than those of the extracts for both O_2_^•−^ and H_2_O_2_.

#### 2.5.3. DNA Mutagenic Damage Protection of *F. punensis* Extracts

*F. punensis* extracts inhibited both point mutations of the “framshift” type, detected by the *S. Typhimurium* TA98 strain, and point mutations of the base pair substitution type, detected by the *S. Typhimurium* TA100 strain, induced by the mutagen 4-NDP (Table 3).

The extracts showed a strong antimutagenic effect at the concentration of 2000 µg GAE/plate with % MI between 42.54 and 68.82. It is important to note that even the concentration of 1000 µg GAE/plate of UAE and MAE extracts showed a strong effect on the TA98 (% MI = 53.73 and 41.01, respectively). In the case of the TA100 strain, the antimutagenic effect was only detected at the highest concentration evaluated. However, *F. punensis* extracts do not exceed the antimutagenic activity of green tea (*C. sinensis*) (Table 3).

#### 2.5.4. Cytotoxic/Anti-Proliferative Activity of *F. punensis* Extracts

To evaluate the chemotherapeutic potential of *F. punensis* extracts obtained in a conventional and non- conventional way, the cytotoxic/anti-proliferative properties were analyzed on the cellular cell line of human melanoma, SKMEL-28.

The results obtained are shown in Table 4 as lethal concentration 50 (LC_50_), defined as the concentration of the extracts required to produce 50% lethality in tumor cells. In the MTT reduction assay, a dose-dependent activity with LC_50_ values between 14 and 18 µg GAE/mL was observed. Also, in the evaluation of the cytotoxic potential using the Neutral Red coloring assay, the LC_50_ values obtained were between 40 and 60 µg GAE/mL. In both experiments, no differences between the different extraction methods were observed. These values are found in the range of the LC_50_ exhibited by quercetin.

### 2.6. Toxicological Evaluation of F. punensis Extracts

The genotoxicity evaluation and acute toxicity of *F. punensis* extracts were evaluated by using the prokaryote *Salmonella* Typhimurium and the crustacean *Artemia salina* as well as the nematode *Caenorhabditis elegans*.

The mutagenicity of *F. punensis* macerations was evaluated by using the Ames test. In this test, the number of revertant colonies per plate induced for all assayed concentrations of all the samples did not exceed the values of revertant colonies of the negative control (15 ± 4 revertant colonies/plate for *Salmonella* Typhimurium TA98 strain and 91 ± 6 revertant colonies for *Salmonella* Typhimurium TA100 strain), which indicates that the extract of *F. punensis* would not induce mutations of the type of change in the open reading frame, detected with strain TA98, or of the base pair substitution detected with strain TA100. Table 5 shows the LC_50_ values for each of the *F. punensis* extracts against *A. salina* and *C. elegans*. The viability of the control group was 100% for both trials. The LC_50_ values of the extracts ranged between 90 and 113 µg GAE/mL against *A. salina*, and no alterations were observed in the swimming of the crustacean larvae. For *C. elegans* the LC_50_ values were between 47.85 and 54.73 µg GAE/mL, without presenting statistical differences between them.

## 3. Discussion

TPC and F content, as well as the percentage of the scavenging of the ABTS^•+^, were the response variables monitored in the extractions performed employing UAE, MAE and conventional maceration. The extracts obtained by UAE exhibited an increased concentration of TPC and F with an increasing solid/liquid relation factor. The same pattern was observed with the factor time, which can be explained considering that diffusion is a time-dependent phenomenon [15]. Meanwhile, the extraction yields of the phytochemicals decreased with increasing extraction amplitude, particularly significant for F (Figure 1). The mechanical effect of increasing amplitude enhances the rupture of plant cell walls, thereby improving extraction efficiency during ultrasonication [16]; however, it also increases the temperature of the process leading to potential thermal degradation of the compounds present due to the generated heat and cavitation. The solid/liquid ratio factor applied in MAE-derived extracts notably and positively influenced both extraction efficiency and antioxidant activity. This aligns with findings presented by Banožić et al. [17], who similarly optimized phenolic compound extraction from different parts of a Solaneaceae family member, the tobacco plant, using RSM. While previous studies have optimized extraction methods for Solanaceae family species, research employing MAE and RSM for genus Fabiana, specifically *F. punensis*, remains unreported. Consequently, the present study is an unprecedented contribution to the optimization of the extraction of this species.

In addition, when comparing the extraction yield of the three methods at their different levels, UAE achieves similar concentrations of TPC and F as conventional maceration, but in a significantly shorter time, while the concentration of soluble principles increases with higher extraction amplitude. MAE, despite yielding lower TPC and F, showed extraction rates 6000 times faster than traditional maceration, emphasizing the time efficiency of non-conventional methods.

The structural changes observed in SEM images demonstrate that the degree of structural alteration in plant matrix tissues varies with the extraction method employed, potentially impacting permeability and yield. Such insights should be crucial for optimizing extractive techniques.

Therefore, UAE and MAE processing could be more convenient approaches for further ramping up production, thanks to its potential in industry, because of their low operating costs and high yields. However, the boosting of these methods is at an early stage and poses many different challenges for the future, including additional optimization work on the UAE and MAE [18,19]. In this regard, Talmaciu et al. [20] compared the cost of UAE, MAE and supercritical fluid extraction (SFE) for polyphenol extraction and concluded that UAE provided the lowest capital cost.

Concerning the anti-inflammatory activity, the extracts of *F. punensis* from the Monte region demonstrate good inhibitory action against LOX enzymes. Indeed, the potency was higher than that previously reported for hydroalcoholic extracts of *F. punensis* from the Puna region [3], and for other plant species that grow in arid and semiarid environments of northwest Argentina [21,22]. In addition, this is the first time that the activity of *F.* punensis extracts on the XO enzyme has been reported. Apart from this NOA species, others belonging to the Solanaceae family from arid environments, such as *Hyoscyamus reticulatus* L., also succeeded in inhibiting this enzyme [23].

Reactive oxygen species, such as hydrogen peroxide or superoxide anion radical, can drive cellular proliferation or differentiation by activating the signaling system. In a biological system, it is produced by a variety of oxidizing enzymes, including superoxide dismutase. However, oxidative stress and inflammatory responses are caused by abnormal reactive oxygen species buildup, with these reactions being linked to pathological disorders such as cancer, diabetes, and cardiovascular illnesses [24]. In biological research, controlling reactive oxygen species production with plant antioxidants is of great interest. This is the first time that the reactive oxygen species scavenging capacity of *F. punensis* of the Monte region has been reported. Other medicinal plants that grow in the Monte region were also capable of scavenging these radicals but with higher concentrations required [21].

Conventional and non-conventional macerations of *F. punensis* protected DNA from agents capable of producing point mutations of the base pair substitution type and, to a greater extent, mutations of the frameshift type. Extract obtained by UAE showed the best activity and may be related mainly to the antioxidant properties exerted by the phytocomplexes. There are no previous reports on the antigenotoxic activity of hydroalcoholic extracts of *F. punensis*.

As for the chemotherapeutic potential of the *F. punensis* extracts evaluated, these are the first data about the cytotoxic activity of hydroalcoholic extracts of the Fabiana genus on tumor cell lines. This experimental evidence foresees a promising future for these extracts as antitumor agents, based on their cytotoxicity in neoplastic cell lines and the basic mechanisms that underlie that effect. This property makes *F. punensis* extracts potential antitumor agents or chemotherapeutic agents.

Chemical patterns obtained using HPLC-DAD indicate that *F. punensis* extracts are a source of polyphenolic compounds. The main constituents found in the extracts were phenolic acids and flavonoids, and the flavonoid apigenin was identified in the extracts.

The most extensively studied plant secondary metabolites are polyphenols; between them, flavonoids, phenolic acids, stilbenes, lignans and others have been studied. Flavonoids are the more abundant polyphenols present in plants and epidemiological studies suggest their role in the prevention of disease. Apigenin is a non-mutagenic flavonoid found in several plants [25,26]. A high number of studies carried out over the years have indicated that apigenin has many interesting pharmacological activities. As an example, its properties as an antioxidant are well known, and it can also be a therapeutic agent to overcome diseases like inflammation, autoimmune, and neurodegenerative diseases, as well as several types of cancers including melanoma and others skin diseases [27]. Additionally, apigenin exhibits anti-mutagenic and antiviral effects [28]. Thus, the presence in the extracts of phenolic compounds, such as apigenin, can explain, at least in part, the biological effect observed in *F. punensis* extracts.

In addition, toxicity test results indicate that, at the concentrations at which the *F. punensis* extracts tested for exhibit biological activity, they should not be toxic, suggesting that their intake in adequate doses would be safe.

## 4. Materials and Methods

### 4.1. Sample Collection

Aerial parts of *F. punensis* (20 cm-long branches) in flowering stage were collected in Abra del Infiernillo, (26°36′41.7″ S 65°50′22.4″ W) from Monte region (Tucumán, Argentina) during January 2018. The plant material was identified by the botanist Dr. Ana Soledad Cuello. Voucher specimens were deposited in the Fundación Miguel Lillo Herbarium (LIL 439).

### 4.2. Plant Extract Preparation

The collected plant material was dried in a forced air oven until weight was consistent, at a temperature of 40 °C with controlled humidity. Once dried, the plant material was ground in a grinder (Numak F100, Numak, London- United Kingdom). The obtained powder was used to prepare the extracts.

### 4.3. Conventional Extraction Method

For conventional macerations, 1 g of plant material was weighed and macerated with 20 mL of 80° ethanol; this mixture was placed in a shaker at 40 cycles per minute, at 40 °C for seven days, in darkness. Then, the mixture was filtered by using Whatman N° 1 paper, applying a vacuum. Finally, they were centrifuged at 5000 rpm for 3 min to eliminate any suspended particles and brought to a final volume of 20 mL with 80 ° ethanol.

### 4.4. Non-Conventional Extraction Methods

#### 4.4.1. Experimental Design and Statistics Analysis

Experiments for non-conventional extractions of *F. punensis* were performed by using UAE and MAE. The experimental design was constructed with Design Expert software (free trial version 13.0.0, StatEase Inc., Minneapolis, MN, USA) by using a spherical Central Composite Design (CCD) to facilitate studies that focus on the combined effect of independent variables on the desired responses or dependent variables.

The range of the independent variables was established based on the alpha value (1.68), and the range of minimum and maximum parametric values of the process included three levels of each factor, that is to say, 25, 50 and 100% amplitude for UAE extraction and 300, 500 and 700 W Power for MAE; 10, 20 and 30 min for UAE and 30, 60 and 90 s for MAE; and 0.25, 0.50 and 1 g of plant material per 20 mL of solvent for both non-conventional extraction methods in terms of +1, 0 or −1 levels. The range of the factors was selected based on an extensive literature review. The upper limit of the extraction amplitude, power and time was chosen to avoid the degradation of bioactive compounds sensitive to temperature increases during the process. Both the yield of total phenolic compounds (TPC) and flavonoids (F), and the percentages of ABTS radical cation (ABTS^+^) scavenging were monitored for each extract obtained from each treatment. Once these values were obtained, appropriate models were fitted to perform the optimization of the extraction processes based on the RSM model. Several alternative models, such as linear, two-factor, interaction, quadratic and cubic models had been considered by the software. Among them, the best model was identified based on the *p*-value (lowest desirable) and F-value (highest desirable) of the models.

#### 4.4.2. Ultrasound Assisted Extraction (UAE)

UAE of *F. punensis* was performed in an open system ultrasonic bath (Hielscher, UP200). The amount of plant material and solvent (80° ethanol) for each extraction was chosen according to the CCD design (Supplementary Table S1). The extraction cycles involved 10 s running and 5 s rest throughout the stipulated time; then, the extracts were filtered by using Whatman N° 1 filter paper, applying a vacuum. Once centrifuged, they were brought to a final volume of 20 mL and stored in a freezer for further use. Each extraction was performed in duplicates.

#### 4.4.3. Microwave Assisted Extraction (MAE)

Microwave-assisted extraction (MAE) of *F. punensis* was performed in a domestic microwave oven adapted for condensation of vapors generated during the extraction processes (BGH-B228DS). The amount of plant material and solvent for each extraction was chosen as indicated by the CCD (Supplementary Table S3). After the MAE treatment, the extracts were filtered by using Whatman N° 1 filter paper in a Büchner funnel and then were brought to a final volume of 20 mL. The extract was stored at 4 °C until further use, and each extraction was performed in duplicate.

### 4.5. Chemical Analysis

#### 4.5.1. Quantification of the Yield Extraction

For the determination of the extraction yield in dry weight (DW), the solvent was totally evaporated in a rotary evaporator (BÜCHI R-110). The remaining fraction was frozen at −20 °C and then freeze-dried (freeze-dryer L-M10-A-E50-CRT, RIFICOR) to remove the water content to obtain a solid residue. By weighing the difference before and after the freeze-drying process, the milligrams of DW contained in 1 mL of the macerations and in 1 g of plant material were determined as well as the milligrams of DW contained per gram of plant material.

#### 4.5.2. Total Phenolic Compound (TPC) Content

Total phenolic compounds content was determined by the Folin–Ciocalteau reagent (Folin—Cicolteau reagent, Merck, Darmstadt, Germany) [29]. A solution of gallic acid (1 mg/mL) was used to develop a calibration curve. The measurements were repeated in triplicate and results were expressed as µg equivalents of gallic acid per mL of extract (µg GAE/mL).

#### 4.5.3. Total Flavonoid (F) Content

Testing for total flavonoid content was performed according to the method of Woisky and Salatino [30] by using AlCl_3_. A quercetin solution (1 mg/mL) was used as a reference compound for the construction of a standard curve and the results were expressed as mg equivalents of quercetin per mL of extract (mg QE/mL).

### 4.6. Phenolic Compounds Profile. HPLC-DAD

Chromatographic profiles of ethanolic extracts of Fabiana species were performed. To obtain the chromatographic profiles, an HPLC equipment with Waters 1525 binary pump system was used, using a C18 XBridgeTM column (5 μm, 4.6 × 250 mm, Waters Corporation, Milford, MA, USA), with a manual injector with injection valve and 20 μL loop (Rheo-dyne Inc., Cotati, CA, USA) and a Waters 2998 UV-Visible detector by diode array (PDA). For sample injection, in all cases, the samples were prepared by resuspending the DW of each sample in methanol at a final concentration of 20 mg DW/mL. The solvent system for the separation of components from extracts was composed of solvent A (0.1% acetic acid in water) and solvent B (0.1% acetic acid in methanol), (conditions: 10–57% B from 0 to 45 min, 57–100% B from 45 to 60 min, and kept at 100% B from 60 to 65 min). The flow rate was set at 0.5 mL/min. Data collection was performed with Empower TM 2 software. The presence of phenolic compounds in extracts was confirmed by UV spectrometry (220–500 nm) in comparison with the standard compounds. The relevant chromatograms were extracted at 330 nm.

### 4.7. Scanning Electron Microscopy

For scanning electron microscopy (SEM), 1 g of the residual plant material of *F*. *punensis* subjected to 30 min extraction at the three amplitude levels for UAE and at 90 s at the three power levels for MAE were coated with gold (Fine Coat Ion Sputter JEOL JFC-1100). The same analysis was performed in dried plant material hydrated in physiological solution for comparative purposes as on the residual material from the conventional maceration of this species. Scanning electron microscopy (SEM) of the gold-coated samples was performed by using a ZEISS SUPRA-55 VP field emission scanning electron microscope at the Centro Integral de Microscopía Electrónica (CIME), CONICET-UNT.

### 4.8. In Vitro Biological Activities

#### 4.8.1. Antioxidant Activity

##### Scavenging of the Radical Cation ABTS (ABTS^•+^)

The technique described by Re et al. was followed [31]. The assay was performed in a microplate (Multiskan GO, Thermo Fisher Scientific Inc., Waltham, MA, USA) and the decrease in the absorbance was recorded at one minute and six minutes after the start of the reaction, and then the percentages of decolorization were calculated. Ethanol 80° was used as negative control. Results are expressed as SC_50_ values (μg GAE/mL), which denotes the concentration required to scavenge 50% of ABTS^•+^.

##### Hydrogen Peroxide Scavenging

The technique described by Fernando and Soysa was followed [32]. The reaction was incubated, and product formation was determined by measurement of the absorbance at 504 nm in a microplate reader (Thermo Fisher Scientific, Multiskan GO). Ethanol 80° was used as a negative control and quercetin was used as a positive control. Results are expressed as SC_50_ values (μg GAE/mL).

##### Superoxide Anion Scavenging

The method of Valentao et al. [33] was followed, with some modifications. In this assay, radicals were generated in a reaction mixture with 40 μL of 20 mM sodium phosphate buffer (pH 7.4), 4 μL of 2 mM NADH, 30 μL of 0.5 mM nitrotetrazolium blue (NBT) and 40 μL of 60 μM phenazine-metasulfate (PMS). This mixture was contacted with different concentrations of the samples evaluated up to a concentration of 100 μg GAE/mL. Etanol 80° and quercetin were employed as negative and positive controls, respectively. After 20 min of reaction, absorbance was measured at 560 nm by using a microplate reader (Thermo Fisher Scientific, Multiskan GO). Results are expressed as SC_50_ values (μg GAE/mL).

#### 4.8.2. Anti-Inflammatory Activity

##### Lipooxygenase (LOX) Inhibition

The inhibitory properties of the extracts on the activity of LOX were determined following the protocol of Taraporewala and Kauffman [34]. The quantity of hydroperoxides produced from linoleic acid was determined by measuring the absorbance at 234 nm on a microplate reader (Thermo Fisher Scientific, Multiskan GO). Naproxen (25 μg/mL), a commercial anti-inflammatory drug, was used as a positive control. The inhibition percentage of the different extracts (12.5 μg GAE/mL) compared to the 100% activity control were obtained according to the following formula, where A is the absorbance measurement at 234 nm.

%_inhibition_= (A_control_ − A_sample_)/A_control_ × 100%

##### Xanthine Oxidase (XO) Inhibition

The technique described by Cos et al. [35] was followed, with some modifications. The effect of 25 μg GAE/mL of the extracts on xanthine oxidase enzyme activity was determined spectrophotometrically. Each sample was added up to 171 μL of 200 mM sodium phosphate buffer (pH 7.5), and 30 μL of the enzyme 0.1 U/mL was dissolved in buffer (EC 1.17.3.2, Sigma Aldrich, Darmstadt, Germany) and incubated at 25 °C for 15 min. The reaction was initiated with the addition of 60 μL of 1 mM xanthine dissolved in the same buffer. The reaction mixture was incubated at 25 °C for 30 min and product formation was recorded by measuring absorbance at 290 nm on a microplate reader (Thermo Fisher Scientific, Multiskan GO). DMSO was used as a negative control and Allopurinol (1 μg/mL) as a positive control. The percentages of enzyme inhibition produced by the different extracts compared to the 100% activity control were calculated according to the following formula, where A is the value of absorbance obtained at 290 nm:

%_inhibition_= (A_control_ − A_sample_)/A_control_ × 100%

#### 4.8.3. DNA Mutagenic Damage Protection

The Ames test [36] was used to evaluate the potential of *F. punensis* extracts to protect cellular DNA from damage produced by a mutagenic agent. Aliquots of 2 mL of semi-soft agar (6 mg/mL agar plus 5 mg/mL NaCl), supplemented with 0.5 mM D-biotin (Sigma-Aldrich) and 0.5 mM L-histidine (Sigma-Aldrich) were prepared, and 100 μL of bacterial culture grown in Mueller–Hinton broth (Britania, Buenos Aires, Argentina) for 14–16 h at 37 °C were added; also, 100 μL of the different concentrations (500–2000 μg GAE/plate) of the samples were added, as well as the tested mutagenic agent 4-NPD (10 μg/plate). DMSO was used as a negative control of the assay. Green tea infusion was included as positive control (1000 µg/mL). The resulting mixture was plated on plates with minimal medium (without histidine) prepared according to the technique. The plates were incubated at 37 °C for 48 h and the number of revertant colonies per plate was counted, and the percentage inhibition of mutagenicity (%IM) produced by each sample with respect to the mutagen plate was calculated according to the following equation:

%IM = 100 − (n° Rev_extract_ × 100 ÷ n° Rev_mutag._)

where n° Rev_extract_ corresponds to the number of revertant colonies/plate on plates with extract and n° Rev_mutag._ corresponds to the number of revertant colonies/plate induced by the mutagen. To indicate the antimutagenic power of a sample it was considered that %IM values higher than 25% would denote weak mutagenic inhibition (and were not recognized as a positive result), 25–40% moderate, and higher than 40% mutagenic inhibition denotes a sample with strong antimutagenic potency. Samples were assayed three times per duplicate.

#### 4.8.4. Cytotoxic/Anti-Proliferative Activity on Tumor Cell Line

##### Cell Culture

Human melanoma cell lines (SKMEL 28) were obtained from the Multidisciplinary Institute of Biology (IMBICE, Buenos Aires, Argentina). The cells were cultured in DMEM-high glucose medium (New Cell, Córdoba, Argentina) supplemented with 10% fetal bovine serum (NatoCor, Córdoba, Argentina), and 100 U/mL penicillin and 100 μg/mL streptomycin (Sigma-Aldrich) at 37 °C in a humidified 5% CO_2_ atmosphere. Experiments were performed by using cells between the 3rd and 5th passage after thawing.

##### MTT Colorimetric Assay

The antiproliferative potential of *F. punensis* extracts was evaluated by the MTT reduction assay [37] against the SKMEL-28 cell line. Cell viability was quantified by the ability of live cells to reduce the yellow dye 3-(4,5-dimethylthiazol-2-yl)-2,5-diphenyltetrazolium bromide to a purple formazan product. The cells were seeded in 96-well plates (2.5 × 10^4^ cells/well) and 10 μL of each extract dissolved in DMSO was added at different concentrations (3.12–50 μg GAE/mL). Quercetin was included as a positive control. After 24 h of treatment, the supernatant was replaced with DMEM-high glucose containing MTT (0.5 mg/mL). The results were expressed as LC_50_ (concentration in μg GAE/mL of the extracts required to produce 50% tumor cell lethality). Samples were assayed four times in triplicate.

##### Neutral Red Colorimetric Assay

The viability assay using Neutral Red is based on the ability of live cells to incorporate and bind Neutral Red stain and the protocol followed was proposed by Borenfreud et al. [38]. 2.5 × 10^4^ cells/well were seeded and the evaluated extracts were added at different concentrations (3.12–50 μg GAE/mL) dissolved in DMSO. Quercetin was included as a positive control. The percentages of cell viability were calculated and the LC_50_ (μg GAE/mL) was obtained by plotting these percentages vs. the concentrations evaluated. Samples were assayed four times in triplicate.

### 4.9. Toxicological Evaluation

#### 4.9.1. Evaluation of Genotoxicity Using Bacterial Cultures: Ames Assay

The mutagenicity assay by using *Salmonella* Typhimurium was performed according to the technique of Maron and Ames [36]. Between 500 and 2000 μg GAE/plate of the different extracts of *F. punensis* were evaluated. DMSO was used as a negative control for the assay and the mutagen 4-nitro-o-phenylenediamine (4-NPD) (10 μg/plate) was used as a positive control. The resulting complete mixture was plated on minimal agar plates. The plates were then incubated at 37 °C for 48 h and the number of revertant colonies per plate (N° revertants/plate) was counted. An extract is considered mutagenic if the number of revertants per plate is more than twice the number of revertant colonies present on the control plate. The control plate gives the frequency of spontaneous revertants. Samples were assayed twice in duplicate, and the results were expressed as the number of revertant colonies/plate.

#### 4.9.2. Toxicity Evaluation by Using *Artemia salina* as a Model

The lethality bioassay against the crustacean *Artemia salina* was used. The assay was performed on a microplate, employing artificial seawater, prepared according to the formula of Perez and Gillin [39]. Early larval stages were used for testing by evaluating different concentrations of the extracts (up to 200 μg GAE/mL). Subsequently, 100 μL of seawater containing 10 nauplius larvae was added. A solution of K_2_Cr_2_O_7_ was used as a positive control and DMSO as a negative control. After an exposure time of 24 h, live and dead nauplii, i.e., those that showed no movement and were at the bottom of the well were counted by using a binocular magnifying glass (10×, Nikon, Tokyo, Japan) and the percentage mortality was calculated for each treatment. The results were expressed as LC_50_ (concentration in μg GAE/mL of the extracts required to produce 50% nauplii lethality) Samples were assayed twice in triplicate.

#### 4.9.3. Toxicity Evaluation Using the Nematode *Caenorhabditis elegans*

For toxicity assays, sterile 24-well polycubes were used according to Solis and Petrascheck [40], with some modifications. Synchronized nematodes were resuspended in a M9 buffer. 20 μL of the nematode solution containing 50 individuals were transferred to each well previously seeded with the different samples’ concentrations (12.5–100 μg GAE/mL) dissolved in DMSO. It was shaken for two minutes and incubated at 25 °C. Nematodes that did not show movement at the tail, head or pharynx level when observed under magnification were considered dead. The LC_50_ (μg GAE/mL) was determined, defined as the concentration of the extracts killing 50% of the nematode population. Samples were assayed twice in triplicate.

### 4.10. Statistical Analysis

The free trial of Design-Expert software version 13.0.0 was used to perform the optimization experiment and modeling. R Studio Team (2020) software was used for statistical analysis. A one-way analysis of variance (ANOVA) test was used to assess the significance of the influence of independent variables and interactions followed by the Tukey post hoc test. All results were expressed as the mean ± standard deviation. At *p*-value ≤ 0.05, differences between samples were considered statistically significant.

## 5. Conclusions

The present work is the first study to optimize the extraction conditions to maximize the yield of bioactive total phenolic compound (TPC) and flavonoids (F) of *F. punensis* aerial parts by using non-conventional extraction methods, namely ultrasound-assisted extraction, UAE, and microwave-assisted extraction, MAE. The developed RSM models allow for predicting the optimized extraction conditions for both UAE and MAE. All extracts exhibited comparable biological attributes, and the ultrasound-derived extract stood out for its anti-inflammatory and antimutagenic characteristics. HPLC-DAD patterns obtained indicates that *F. punensis* extracts are a source of polyphenolic compounds. The results of the work are important in the development of efficient and sustainable methods for obtaining bioactive compounds from *F. punensis*. for the prevention of chronic degenerative diseases associated with oxidative stress, inflammation, and DNA damage.

## Figures and Tables

**Table 1:** Comparison of the predicted and experimental values of the yield of total phenolic compounds (TPC) and flavonoids (F), and scavenging activity of ABTS^•+^ of *F. punensis* under optimal conditions by using UAE (ultrasound-assisted extraction) or MAE (microwave assisted extraction).

UAE						
Amplitude (%)	Time (min)	Solid/Liquid Ratio (g/mL)	Phytochemicals	Predicted Value	Experimental Value	Prediction Error (%)
25	30	1:20	TPC (µg GAE ^1^/mL)	1275.33	1183.85 ± 1	7 ± 0.43
			F (µg QE ^2^/mL)	172.03	162.31 ± 0.2	5 ± 0.39
			MAE			
**Power (W)**	**Time** **(seg)**	**Solid/Liquid Ratio** **(g/mL)**	**Phytochemicals**	**Predicted Value**	**Experimental Value**	**Prediction Error (%)**
700	90	1:20	TPC (µg GAE ^1^/mL)	562.67	584.97 ± 21	4
			F (µg QE ^2^/mL)	129.88	133.60 ± 3	2
			ABTS^•+^ (%)	48.14	51.9 ± 4.7	7

Values are presented as mean ± standard deviation (n = 3). GAE ^1^: gallic acid equivalents. QE ^2^: quercetin equivalents. TPC: Total phenolic compounds. F: flavonoids.

**Table 2:** Comparative analysis of total phenolic compounds (TPC), flavonoids (F) and ABTS^•+^ scavenging and DW content using conventional, UAE-optimized and MAE-optimized extraction methods.

	Conventional	UAE-Optimized	MAE-Optimized
TPC [µg GAE ^1^/mL]	1308 ± 9.2 ^A^	1348.03 ± 10.2 ^A^	524.8 ± 35.1 ^B^
F [µg QE ^2^/mL]	221 ± 19.8 ^A^	212.36 ± 17.2 ^A^	144.08 ± 12.3 ^B^
ABTS^•+^ [%]	50 ± 4.6 ^B^	74 ± 5.7 ^A^	57 ± 4.8 ^B^
DW [mg/mL]	9.94 ± 0.8 ^B^	17.4 ± 1.2 ^A^	9.8 ± 0.7 ^B^
Time [days/min/seg]	7 days	30 min	90 seg
Volume of solvent	20 mL	20 mL	20 mL
Plant material	1 g	1 g	1 g

Values are presented as mean ± standard deviation (n = 3). UAE: ultrasound-assisted extraction. MAE: microwave assisted extraction. TPC: Total phenolic compounds. F: flavonoids. DW: dry weight. GAE ^1^: gallic acid equivalents QE ^2^: quercetin equivalents. Different letters represent statistically significant differences at *p* ≤ 0.05 between two columns, according to Tukey’s test

**Table 3:** Percentage of mutagenic inhibition (% MI) exerted by extracts from *F. punensis* against the mutagen 4-NDP.

Extraction Method	TPC/Plate [µg GAE ^1^/Plate]	Percentage of Mutagenic Inhibition (% MI)	
		TA98	TA100
UAE	500	24.63 ± 3.84 ^D^	13.11 ± 1.31 ^D^
	1000	53.73 ± 8.34 ^B^	17.84 ± 0.78 ^D^
	2000	68.82 ± 1.72 ^B^	48.35 ± 4.83 ^BC^
MAE	500	14.92 ± 5.10 ^D^	19.93 ± 1.4 ^D^
	1000	41.01 ± 646 ^C^	28.13 ± 3.1 ^D^
	2000	66.37 ± 5.55 ^B^	42.54 ± 4.51 ^C^
Conventional	500	6.32 ± 1.90 ^E^	17.65 ± 2.3 ^D^
	1000	32.90 ± 5.37 ^C^	11.36 ± 1.13 ^D^
	2000	55.43 ± 7.18 ^B^	52.36 ± 7.4 ^B^
C (+) green tea	1000	74.10 ± 1.80 ^A^	69.01 ± 2.25 ^A^

Results are expressed as the mean of six determinations ± standard deviation. Values with a common letter for the same column are not statistically different (Tukey’s test *p* ≤ 0.05). UAE: ultrasound-assisted extraction. MAE: microwave assisted extraction. Green tea was included as antimutagenic positive control, C (+). TPC: Total phenolic compounds; ^1^ GAE: gallic acid equivalents.

**Table 4:** Effect of *F. punensis* extracts on the viability of tumor cells evaluated by the MTT reduction assay and the Neutral Red assay.

	MTT Assay	Neutral Red Assay
Extraction Method	LC_50_ (µg ^1^ GAE/mL)	LC_50_ (µg ^1^ GAE/mL)
UAE	18.0 ± 2.1 ^A^	60.0 ± 8.8 ^A^
MAE	14.0 ± 1.7 ^A^	40.0 ± 8.1 ^A^
Conventional	16.0 ± 2.0 ^A^	42.0 ± 7.7 ^A^
Quercetin	15.1 ± 1.8 ^A^	52.3 ± 6.0 ^A^

LC_50_: concentration of the extracts of *F. punensis* required to produce 50% tumor cell lethality. UAE: ultrasound-assisted extraction. MAE: microwave assisted extraction. ^1^ GAE: gallic acid equivalents. Quercetin was used as positive control. Values are expressed as the mean of three determinations ± standard deviation. Values with a common letter represent statistically non-significant differences (Tukey’s test: *p* ≤ 0.05).

**Table 5:** Toxicity of *F. punensis* extracts on *A. salina* nauplius and *C. elegans* nematodes expressed as percentage of lethality.

	*A. salina*	*C. elegans*
Extraction Method	LC_50_^a^ (µg ^1^ GAE/mL)	LC_50_^b^ (µg ^1^ GAE/mL)
UAE	90.00 ± 7.50 ^A^	47.85 ± 5.60 ^A^
MAE	112.59 ± 8 ^A^	49.00 ± 6.10 ^A^
Conventional	113.77 ± 11 ^A^	54.73 ± 4.30 ^A^

LC_50_: Concentration of the extracts required to produce 50% lethality of the nauplii (^a^) or nematodes (^b^). UAE: ultrasound-assisted extraction. MAE: microwave assisted extraction. ^1^ GAE: gallic acid equivalents. Values are presented as the mean ± standard deviation. Values with a letter in common for the same column are not significantly different (Tukey’s test, *p* ≤ 0.05).

## Data Availability

Data is contained within the article or supplementary material.

## Supplementary Materials

The following supporting information can be downloaded at: https://www.mdpi.com/article/10.3390/molecules29153578/s1, Table S1: Response surface methodology of a three-variable three-level CCD and corresponding response values of total phenolic compounds (TPC), flavonoids (F) and scavenging activity of ABTS^•+^ of *F. punensis* extracts using UAE as extraction method.; Table S2: Analysis of variance for fitted regression model for the extraction of total phenolic compounds (TPC) and flavonoids (FF) using UAE as extraction method. Table S3: Response surface methodology of a three-variable three-level CCD and corresponding response values of total phenolic compounds (TPC), flavonoids (FF) and scavenging activity of ABTS^•+^ of *F. punensis* extracts using MAE as extraction method. Table S4: Analysis of variance for fitted regression model for the extraction of total phenolic compounds (TPC), flavonoids (F) and the scavenging activity of ABTS^•+^ using MAE as extraction method.
