# Supplementary material for: Optimization of Phenolic Compounds Extraction from Aerial Parts of Fabiana punensis S. C. Arroyo by Ultrasound- and Microwave-Assisted Extraction
Source: Molecules. 2024 Jul 29;29(15):3578. doi: 10.3390/molecules29153578 (PMC11313804; doi:10.3390/molecules29153578)
Supplement: Supplementary file 1 [file molecules-29-03578-s001.zip › molecules-3015368-supplementary.pdf]

## Supplementary material

**Table S1.** Response surface methodology of a three-variable three-level CCD and corresponding response values of total phenolic compounds (TPC), flavonoids (F) and scavenging activity of $ABTS^{•+}$ of *F. punensis* extracts using UAE as extraction method.

| **Amplitude (A) [%]** | **Time (B) [minutes]** | **Solid/ liquid relation (C) [g/ 20 mL]** | **TPC [µg GAE[1]/mL]** | **F [µg QE[2]/mL]** | **Scavenging activity of $ABTS^{•+}$ [%]** |
|---|---|---|---|---|---|
| -1 | -1 | 1 | 863.11±77.75 | 148.11±13.38 | 94.62±8.52 |
| 0 | 0 | 1.68 | 1231.49±98.51 | 74.08±5.94 | 94.45±7.56 |
| -1 | -1 | 1 | 787.41±66.92 | 136.41±11.61 | 94.39±8.02 |
| 1 | 1 | 1 | 1382.23±124.46 | 143.49±16.97 | 93.67±8.43 |
| 1 | 1 | 1 | 1207.08±108.67 | 151.81±13.74 | 84.31±7.59 |
| 0 | 0 | 0 | 636.22±50.92 | 109.37±8.77 | 83.89±6.71 |
| 0 | 0 | 1.68 | 1231.49±117.08 | 94.49±9.10 | 83.84±7.96 |
| 0 | 0 | 0 | 706.22±63.63 | 110.01±9.83 | 82.11±7.39 |
| -1.68 | 0 | 0 | 692.52±55.49 | 126.29±10.14 | 81.62±6.53 |
| 0 | 1.68 | 0 | 670.08±53.65 | 94.67±7.65 | 81.03±6.48 |
| 0 | -1.68 | 0 | 666.93±53.44 | 105.51±8.47 | 79.02±6.32 |
| 0 | -1.68 | 0 | 683.46±61.57 | 116.36±10.59 | 78.74±7.09 |
| 0 | 0 | 0 | 655.38±59.08 | 110.01±9.95 | 78.12±7.03 |
| 0 | 0 | 0 | 706.22±67.12 | 99.46±9.42 | 75.51±7.12 |
| 0 | 1.68 | 0 | 654.33±52.75 | 91.18±7.55 | 74.44±6.20 |
| -1 | 1 | 1 | 1159.05±113.68 | 172.05±16.93 | 74.19±7.27 |
| -1 | 1 | 1 | 1348.03±117.33 | 172.05±15.35 | 70.65±6.15 |
| 1.68 | 0 | 0 | 610.24±48.29 | 61.41±4.94 | 70.08±5.54 |
| -1.68 | 0 | 0 | 608.27±54.75 | 86.76±7.86 | 67.42±6.07 |
| 1 | -1 | 1 | 691.41±55.37 | 98.35±7.99 | 64.25±5.14 |
| 0 | 0 | -1.68 | 478.43±35.99 | 64.89±4.98 | 59.13±4.43 |
| 1 | -1 | 1 | 688.54±61.30 | 124.08±11.21 | 57.82±5.15 |
| 1.68 | 0 | 0 | 347.64±25.62 | 49.82±3.63 | 50.56±3.64 |
| 0 | 0 | -1.68 | 406.69±26.46 | 45.77±3.25 | 45.07±2.93 |
| -1 | 1 | -1 | 393.31±35.48 | 62.26±5.68 | 44.74±4.03 |
| 1 | -1 | -1 | 416.91±34.22 | 53.86±4.45 | 41.23±3.38 |
| -1 | -1 | -1 | 261.02±19.19 | 48.71±3.64 | 41.04±3.17 |
| -1 | 1 | -1 | 396.39±30.17 | 56.61±4.32 | 40.26±3.05 |
| 1 | -1 | -1 | 416.92±21.53 | 59.24±4.82 | 37.13±3.47 |
| -1 | -1 | -1 | 263.61±18.65 | 53.51±5.15 | 36.93±4.01 |
| 1 | 1 | -1 | 280.74±21.77 | 36.91±4.68 | 34.22±3.21 |
| 1 | 1 | -1 | 278.74±23.48 | 33.33±3.92 | 30.79±3.9 |

Values are presented as mean ± standard deviation (n = 3). GAE[1]: gallic acid equivalents QE[2]: quercetin equivalents.

**Table S2.** Analysis of variance for fitted regression model for the extraction of total phenolic compounds (TPC) and flavonoids (F) using UAE as extraction method.

| Source | Sum of Squares | df[1] | Mean Square | F-value | *p*-value | |
|---|---|---|---|---|---|---|
| **TPC** | | | | | | |
| Block | 8965,31 | 1 | 8965,31 | | | |
| Model | 3,140E+06 | 11 | 2,854E+05 | 109,79 | < 0.0001 | significant |
| A-Amplitude | 1206,15 | 1 | 1206,15 | 0,4640 | 0,5040 | |
| B-Time | 168,74 | 1 | 168,74 | 0,0649 | 0,8016 | |
| C-Solid/ liquid ratio | 6,224E+05 | 1 | 6,224E+05 | 239,43 | < 0.0001 | |
| AB | 2178,09 | 1 | 2178,09 | 0,8378 | 0,3715 | |
| AC | 4464,24 | 1 | 4464,24 | 1,72 | 0,2057 | |
| $A^2$ | 5640,47 | 1 | 5640,47 | 2,17 | 0,1571 | |
| $A^2C$ | 71869,74 | 1 | 71869,74 | 27,65 | < 0.0001 | |
| Residual | 49393,80 | 19 | 2599,67 | | | |
| Lack of Fit | 2508,98 | 3 | 836,33 | 0,2854 | 0,8352 | not significant |
| Pure Error | 46884,82 | 16 | 2930,30 | | | |
| Cor Total | 6.154E+05 | 30 | | | | |
| **F** | | | | | | |
| Block | 588.56 | 1 | 588.56 | | | |
| Model | 42574.32 | 10 | 4257.43 | 32.31 | < 0.0001 | Significant |
| A-Amplitude | 3572.63 | 1 | 3572.63 | 27.11 | < 0.0001 | |
| B-Time | 324.36 | 1 | 324.36 | 2.46 | 0.1332 | |
| C-Solid/ liquid relation | 838.39 | 1 | 838.39 | 6.36 | 0.0207 | |
| AB | 185.20 | 1 | 185.20 | 1.41 | 0.2504 | |
| AC | 399.28 | 1 | 399.28 | 3.03 | 0.0979 | |
| BC | 1389.83 | 1 | 1389.83 | 10.55 | 0.0042 | |
| $A^2$ | 1109.05 | 1 | 1109.05 | 8.42 | 0.0092 | |
| $C^2$ | 2718.91 | 1 | 2718.91 | 20.63 | 0.0002 | |
| $A^2B$ | 812.18 | 1 | 812.18 | 6.16 | 0.0226 | |
| $A^2C$ | 8730.92 | 1 | 8730.92 | 66.25 | < 0.0001 | |
| Residual | 2503.90 | 19 | 131.78 | | | |
| Lack of Fit | 709.96 | 4 | 177.49 | 1.48 | 0.2565 | not significant |
| Pure Error | 1793.94 | 15 | 119.60 | | | |
| Cor Total | 45666.78 | 30 | | | | |

df[1]: degrees of freedom.

**Table S3.** Response surface methodology of a three-variable three-level CCD and corresponding response values of total phenolic compounds (TPC), flavonoids (F) and scavenging activity of $ABTS^{•+}$ of *F. punensis* extracts using MAE as extraction method.

| Power (A) [W] | Time (B) [seg] | Solid/ liquid ratio[1] (C) [g/ 20 mL] | TPC [μg GAE[1]/mL] | F [μg QE[2]/mL] | Scavenging activity of $ABTS^{•+}$ [%] |
|---|---|---|---|---|---|
| -1 | 1 | 1 | 419.29±37.76 | 132.16±11.89 | 57.77±5.20 |
| 1 | -1 | 1 | 479.52±38.42 | 115.62±9.25 | 58.59±4.69 |
| 1 | -1 | -1 | 131.10±11.12 | 35.478±3.02 | 22.31±1.90 |
| -1 | -1 | 1 | 512.20±46.14 | 136.76±12.31 | 56.05±5.404 |
| -1 | -1 | 1 | 506.69±45.68 | 132.45±11.92 | 57.01±5.13 |
| 0 | 0 | 0 | 317.32±24.50 | 70.22±.562 | 39.42±3.15 |
| 1 | 1 | -1 | 196.06±18.67 | 39.89±3.79 | 27.38±2.60 |
| 0 | 0 | 0 | 311.02±28.09 | 86.94±7.82 | 41.31±3.72 |
| 1 | 1 | 1 | 524.803±42.02 | 133.0±10.64 | 57.45±4.60 |
| 1 | 1 | -1 | 196.06±15.76 | 45.588±3.65 | 28.32±2.27 |
| -1 | 1 | 1 | 502.36±40.21 | 132.16±10.57 | 63.98±5.12 |
| -1 | -1 | -1 | 173.22±15.32 | 41.36±3.72 | 20.54±1.85 |
| 1 | -1 | 1 | 532.67±43.73 | 149.44±13.54 | 41.04±3.68 |
| 1 | -1 | -1 | 203.54±19.21 | 45.77±4.35 | 24.49±2.33 |
| 1 | 1 | 1 | 532.67±43.71 | 119.77±9.82 | 68.24±5.60 |
| -1 | -1 | -1 | 175.98±17.25 | 38.05±3.73 | 22.82±2.24 |
| 0 | 0 | 0 | 285.59±24.82 | 89.33±7.77 | 45.91±3.98 |
| 0 | 0 | 0 | 349.05±27.65 | 75.55±5.97 | 36.09±2.85 |
| -1 | 1 | -1 | 146.85±13.02 | 32.90±2.96 | 19.24±1.73 |
| -1 | 1 | -1 | 162.59±13.06 | 34.74±2.78 | 18.28±1.46 |
| 0 | 1.68 | 0 | 435.82±32.74 | 84.92±6.73 | 15.92±1.19 |
| 0 | 1.68 | 0 | 383.46±34.15 | 83.08±7.39 | 16.2±1.44 |
| 0 | 0 | 1.68 | 591.55±41.67 | 145.65±10.01 | 18.05±1.30 |
| 0 | 0 | 1.68 | 577.16±37.58 | 150.73±9.80 | 19.39±1.26 |
| 1.68 | 0 | 0 | 412.20±37.14 | 86.76±7.81 | 12.03±1.08 |
| 1.68 | 0 | 0 | 423.62±34.72 | 84.37±6.92 | 12.95±1.06 |
| 0 | -1.68 | 0 | 395.66±28.90 | 89.33±6.52 | 15.8±1.11 |
| 0 | 0 | -1.68 | 235.43±17.93 | 33.73±2.56 | 5.56±0.45 |
| 0 | -1.68 | 0 | 381.56±22.09 | 60.11±5.62 | 11.63±1.02 |
| 0 | 0 | -1.68 | 227.17±14.56 | 35.01±3.37 | 6.11±0.59 |
| -1.68 | 0 | 0 | 313.38±23.48 | 77.02±5.71 | 14.02±1.25 |
| -1.68 | 0 | 0 | 450.39±30.59 | 94.85±8.34 | 13.39±1.04 |

Values are presented as mean ± standard deviation (n = 3). GAE[1]: gallic acid equivalents QE[2]: quercetin equivalents.

**Table S4**. Analysis of variance for fitted regression model for the extraction of total phenolic compounds (TPC), flavonoids (F) and the scavenging activity of ABTS$^{•+}$ using MAE as extraction method.

| Source | Sum of Squares | df[1] | Mean Square | F-value | p-value | |
|---|---|---|---|---|---|---|
| **TPC** | | | | | | |
| Block | 34740.56 | 1 | 34740.56 | | | |
| Model | 5.579E+05 | 7 | 79706.86 | 77.27 | < 0.0001 | significant |
| A-Power | 3711.93 | 1 | 3711.93 | 3.60 | 0.0710 | |
| B-Time | 0.4133 | 1 | 0.4133 | 0.0004 | 0.9842 | |
| C- Solid/ liquid relation | 1.198E+05 | 1 | 1.198E+05 | 116.09 | < 0.0001 | |
| AB | 3592.92 | 1 | 3592.92 | 3.48 | 0.0754 | |
| AC | 232.74 | 1 | 232.74 | 0.2256 | 0.6395 | |
| $A^2$ | 51.87 | 1 | 51.87 | 0.0503 | 0.8246 | |
| $A^2C$ | 24794.44 | 1 | 24794.44 | 24.04 | < 0.0001 | |
| Residual | 22694.62 | 22 | 1031.57 | | | |
| Lack of Fit | 2134.48 | 6 | 355.75 | 0.2768 | 0.9397 | not significant |
| Pure Error | 20560.14 | 16 | 1285.01 | | | |
| Cor Total | 6.154E+05 | 30 | | | | |
| **F** | | | | | | |
| Block | 135.60 | 1 | 135.60 | | | |
| Model | 43458.46 | 6 | 7243.08 | 94.35 | < 0.0001 | significant |
| A-Power | 0.2884 | 1 | 0.2884 | 0.0038 | 0.9517 | |
| C-Solid/ liquid relation | 9062.83 | 1 | 9062.83 | 118.05 | < 0.0001 | |
| AC | 77.87 | 1 | 77.87 | 1.01 | 0.3244 | |
| $A^2$ | 77.40 | 1 | 77.40 | 1.01 | 0.3258 | |
| $C^2$ | 323.56 | 1 | 323.56 | 4.21 | 0.0516 | |
| $A^2C$ | 696.73 | 1 | 696.73 | 9.08 | 0.0062 | |
| Residual | 1765.68 | 23 | 76.77 | | | |
| Lack of Fit | 177.98 | 7 | 25.43 | 0.2562 | 0.9624 | not significant |
| Pure Error | 1587.71 | 16 | 99.23 | | | |
| Cor Total | 45359.74 | 30 | | | | |
| Scavenging activity of ABTS$^{•+}$ | | | | | | |
| Block | 5422.91 | 1 | 5422.91 | | | |
| Model | 5221.40 | 8 | 652.68 | 42.28 | < 0.0001 | significant |
| A-Power | 2.35 | 1 | 2.35 | 0.1523 | 0.7001 | |
| B-Time | 76.42 | 1 | 76.42 | 4.95 | 0.0366 | |
| C-Solid/ liquid relation | 166.02 | 1 | 166.02 | 10.76 | 0.0034 | |
| AB | 64.40 | 1 | 64.40 | 4.17 | 0.0533 | |
| AC | 60.37 | 1 | 60.37 | 3.91 | 0.0606 | |
| BC | 62.81 | 1 | 62.81 | 4.07 | 0.0561 | |
| $A^2$ | 1.08 | 1 | 1.08 | 0.0699 | 0.7939 | |
| $A^2C$ | 1202.13 | 1 | 1202.13 | 77.88 | < 0.0001 | |
| Residual | 339.60 | 22 | 15.44 | | | |
| Lack of Fit | 40.96 | 5 | 8.19 | 0.4663 | 0.7960 | not significant |
| Pure Error | 298.65 | 17 | 17.57 | | | |
| Cor Total | 10983.91 | 31 | | | | |

df[1]: degrees of freedom.
